# Detection of Increased Expression of Claudin-1 in Triple-Negative Breast Cancer: Analysis and Clinical-Pathological Correlation

**DOI:** 10.7759/cureus.36648

**Published:** 2023-03-24

**Authors:** Abderrahman Ouban, Omar Z Ameer, Ko Jin Quek, Maria A Arafah, Layla Raddaoui

**Affiliations:** 1 Pathology and Molecular Medicine, Alfaisal University College of Medicine, Riyadh, SAU; 2 Pharmaceutical Sciences, Alfaisal University College of Pharmacy, Riyadh, SAU; 3 Family Medicine, Faculty of Biomedical Sciences, Macquarie University, Sydney, AUS; 4 Pathology and Laboratory Medicine, King Saud University, Riyadh, SAU; 5 Oncology, Alfaisal University College of Medicine, Riyadh, SAU

**Keywords:** triple-negative, metastases, claudin-1, beta-catenin, breast

## Abstract

Background

Triple-negative breast cancer (TNBC) is a highly aggressive disease that lacks therapeutic targets and prognostic biomarkers. Claudin-1 is a well-described tight junction protein with prognostic value in many human cancers.

Aims

The need for the discovery of biomarkers of TNBC disease was a major reason for this study. Claudin-1 is a tight junction protein that has shown promising results in the prognosis and management of cancer in general. In the breast, claudin-1 expression and significance have shown variable results, especially in TNBC patients. Our study assessed expression of claudin-1 in a group of TNBC patients, and correlated this expression with clinical-pathological parameters, and with the expression of β-catenin.

Materials and methods

Tissues from a group of 52 TNBC patients were retrieved from the archives of the community hospital. All related information including demographical, pathologic and clinical data were retrieved. Immunohistochemistry assays of a rabbit polyclonal antibody anti-human claudin-1 were applied using the avidin-biotin peroxidase methodology.

Results

A statistically significant majority of TNBC cases positively expressed claudin-1 (81%, χ2=13.705; p<0.001). Most TNBC cases had grade 2 β-catenin expression (77.5%; p<0.001), and positive expression for claudin-1 correlated with that of β-catenin (χ2= 23.757; p<0.001). Claudin-1 and β-catenin expressions within tumour cells shared several features including absent or weakness of membranous expression, and redistribution of both proteins to the cytoplasm of tumour cells, and in some cases to the nuclei of these cells. Claudin-1 expression also correlates with adverse survival outcomes, where only four of 20 claudin-1-positive patients who received neo-adjuvant chemotherapy (NAC) achieved pathological complete response (pCR).

Conclusions

The above presents a complex role of claudin-1 in TNBC patients. In this study, claudin-1 expression was associated with poor prognostic features including invasion, metastases and adverse clinical outcomes. Claudin-1 expression in TNBC correlated with the expression of β-catenin, an important oncogene and a major contributor to the epithelial mesenchymal transition (EMT) phenomenon. Overall, the above results may serve as an impetus for further mechanistic studies to assess the exact role of claudin-1 in TNBC and its possible use in the management of this subset of breast cancer.

## Introduction

Breast cancer is the most common type of malignant tumour among women worldwide. Although research advancements have assisted in the diagnosis and management of breast cancer, it is still the leading cause of mortality for women in many countries [1]. The majority of breast cancer deaths are related to disease progression and metastases. Primary treatment options offered to breast cancer patients may involve surgery, radiation therapy, and adjuvant hormone or chemotherapy. Neoadjuvant chemotherapy (NAC) treatment focuses on treating micro-metastatic disease or breast cancer cells, which may have separated from the breast and the regional lymph nodes, but have not yet grown into an identifiable metastasis [2]. Adjuvant therapy is estimated to be responsible for 35-72% of the reduction in mortality of breast cancer [2]. One important subtype of breast cancer is the primary triple-negative breast cancer (TNBC), which accounts for approximately 16% of all breast cancers worldwide. TNBCs are characterized by a lack of receptor expressions (estrogen receptor [ER]; progesterone receptor [PR]) and lack of expression of human epidermal growth factor receptor 2 (ERBB2 gene, also known as HER2), which is typically seen as a driver of tumourigenesis through amplification and surface cell membrane overexpression, but in rare cases for breast cancer may also be due to an activating mutation(s) [2]. Because TNBCs have fewer easily accessible drug targets, and despite an initially good response to chemotherapy, resistance begins soon after, resulting in recurrence of the tumour with development of multiple metastases [3]. Some studies even estimate a much higher risk of relapse of TNBC cases in the first three years following diagnosis, when compared to ER-positive cases [4]. One likely explanation for development of metastases in breast cancer is the epithelial mesenchymal transition (EMT) phenomenon [5]. In particular, EMT purports that it is the loosening of cell-to-cell connections through the degradation and remodeling of tight junction strands and adherens junction proteins, which allow for the detachment and freer movement of transformed cells. The core proteins of these strands are the claudins, which connect cells side-by-side, thus sealing the intercellular space and protecting the tissues lying beneath it. Claudins exert selective permeability of solutes through their weak and short-lived interactions with nearby same or other claudin proteins regulating paracellular diffusion [6]. Among the more than 27 transmembrane members of this family, claudin-1 features prominently in recent literature, as perhaps the most important member of this family with regard to its relationship with neoplastic transformation [7].

Contradictory results have been reported regarding claudin-1 expression in TNBCs [7]. Some show increased expression, while others show decreased expression of the protein with related clinical-pathological correlations. To investigate this discordant result, we evaluated claudin-1 expression in a group of TNBC patients from our hospital. We also analyze the pattern and intensity of β-catenin’s expression in the same group and correlate this expression with that of claudin-1. β-catenin was selected as a second protein of interest in our TNBC cases because of its close relationship with claudin-1 as two chief components of the Wnt/β-catenin pathway in the development of breast cancer [8], and because of its convergence on the nucleus to combine with a T-cell factor, resulting in increased cellular proliferation in breast cancer [9-10]. This study will address expression of claudin-1 exclusively in a group of TNBC patients and will be added to a long list of studies that have assessed this expression in breast cancer in general [11,12].

## Materials and methods

Tissues samples

The study was performed according to the Declaration of Helsinki and was sanctioned by the Institutional Review Board at King Saud University (E-19-4013). The informed consent was waived in this study because it poses no risk to patients, its retrospective in nature involving analysis of already collected formalin-fixed, paraffin-embedded pathology material, and does not disclose patients’ identities. Records were obtained from the King Khaled University Hospital. Our search criteria specified a five-year period (May 2015 to May 2020). All available pathological and clinical data were collected and reviewed by two independent pathologists (AO and MA). Tissues collected were processed and prepared for either histology staining (hematoxylin-eosin) or immunohistochemistry (these preparations included the followings: PR, ER, HER2/neu; and ERBB2 amplification in HER2/neu equivocal cases). Data collections included age and tumour criteria: size, histological subtype, tumour grade, clinical stage, TNM staging, survival data, ductal carcinoma* in situ *(DCIS) presence, presence of lymph-vascular invasion, ER/PR/HER2/neu expression, and metastatic lymph node disease. Survival data was incomplete and included the following categories: Died; Alive with the Disease (AWD); and Alive without the Disease (AWOD). Because data was incomplete a full survival analysis couldn’t be performed, and the remaining available data was assessed against expression of the claudin-1 protein. The achievement of pathological complete response (pCR) and the presence of residual tumour after administering neo-adjuvant chemotherapy (NAC) were recorded for patients who received this type of treatment. The 4th edition of the Classification of Tumors of the Breast - World Health Organization [13] was used to assign tumour grades. TNM and clinical stages were assigned in accordance with the American Joint Committee on Cancer (AJCC); Cancer Staging Manual, 8th edition [14]. Control samples were obtained via randomly selected ten cases of benign breast lesions.

Immunohistochemistry (IHC) 

Following fixation in formalin, tissue samples were placed in paraffin and were divided into sections that are 5 mm thick. As per manufacturer’s instructions, a rabbit polyclonal antibody anti-human claudin-1 (ab 15098; Abcam, Cambridge, UK); and a rabbit polyclonal anti-human β-catenin (ab16051; Abcam) were diluted (1:300 for claudin-1 and 1:200 for β-catenin) in antibody diluent (Agilent, Santa Clara, CA, USA), using the avidin-biotin peroxidase methodology (Vectastatin Elite ABC kit; Vector Laboratories, Burlingame, CA, USA). Negative controls were done following omission of primary antibody. Positive controls of normal skin were performed for optimization and validation [15].

Staining evaluation 

Claudin-1 IHC

All stained sections were assessed by two pathologists (AO and MA) independently, and an agreement was reached in discordant cases. The evaluating pathologists were blind to the pathological and clinical data. Normal controls and tissues adjacent to cancer or malignant tissue, were compared with normal skin IHC score. Degree of claudin-1 reactivity was scored by both pathologists independently, by applying a semi‑quantitative immunoreactivity scoring system (IRS) as described by Baccelli and colleagues previously [15]. Staining intensity was classified into four grades: 0=no staining; 1=weak; 2=moderate; and 3=strong staining. Percentage of positive cells was categorized into five tiers: tier 0=(0%); tier 1=(1‑10%); tier 2=(11‑50%); tier 3=(51‑80%); and tier 4=(>80%). Staining intensity grade and percentage of positive cells tier were multiplied to obtain the IRS, with a range of 0‑12 for each patient. A case was regarded as positive for claudin-1 if it was allocated an IRS 7-12, and negative with an IRS between 0‑6 [15].

β-catenin IHC

β-catenin expression was described as preserved (Normal) if >80% of the cell membranes of the sample were stained. If these criteria were not met, the sample will be classified as showing reduced, abnormal expression of the protein [16-17] and further classified this abnormal β-catenin expression (ABE) as follows: Grade 1: Loss of membranous staining with cytoplasmic or nuclear staining; Grade 2: Loss of membrane staining with brown granular cytoplasmic staining; Grade 3: Loss of membrane staining with brown nuclear staining, and with or without cytoplasmic staining.

Statistical analysis

The χ2 test was performed to analyze claudin-1 expression in TNBC cases (N=52). This test was also used to assess the correlation between claudin-1 positive cases (N=42) and several pathological, demographics, and clinical attributes including age, sentinel lymph node status, tumour size, clinical stage, TNM staging, nuclear grade, tumour location, DCIS status, survival data, pCR and β-catenin expression. Statistical analysis was performed with version 25.0 of the IBM SPSS Statistics software package (IBM Corp., Armonk, NY, USA). A p value of <0.05 was set to indicate statistically significant difference.

## Results

**Figure 1:**
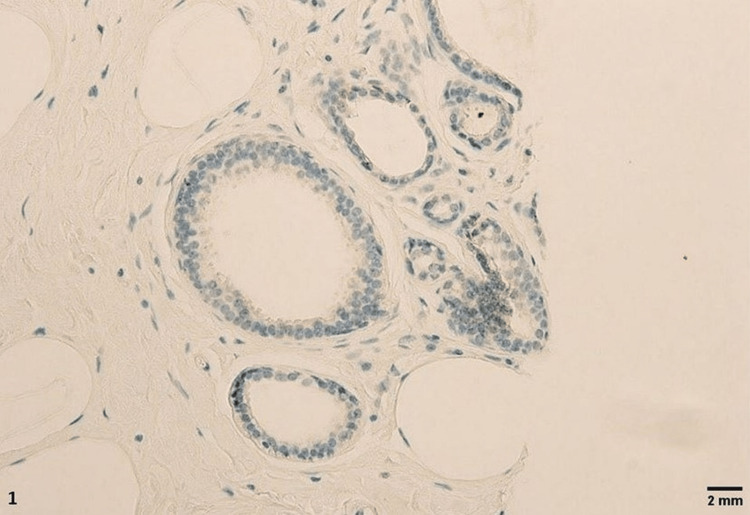
Expression of claudin-1 in normal breast cells

**Figure 2:**
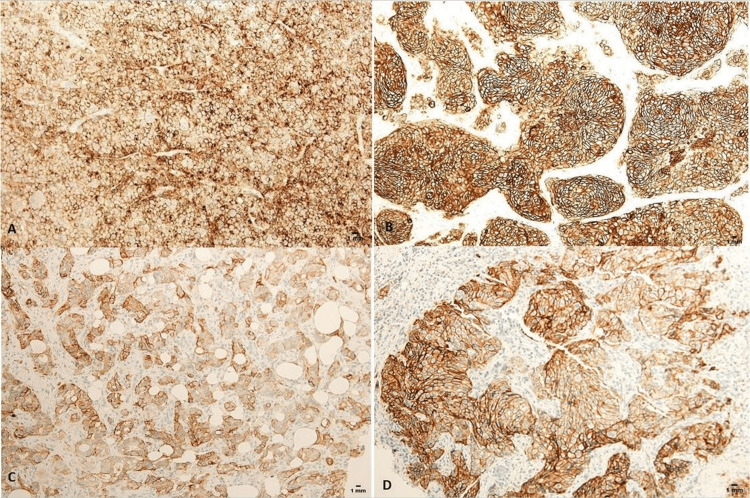
A-D: Expression of claudin-1 in breast cancer cells. All cancer cells in all four tumours (A-D) have robust cytoplasmic expression with or without nuclear one. IRS in all four tumours is 12/12. IRS: immunoreactivity scoring system

**Figure 3:**
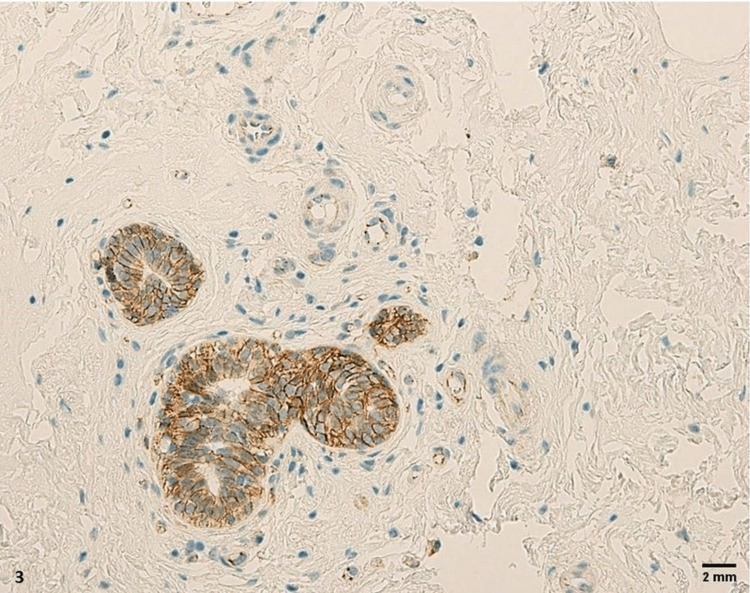
Expression of β-catenin in normal breast cells. The expression is mainly membranous.

**Figure 4:**
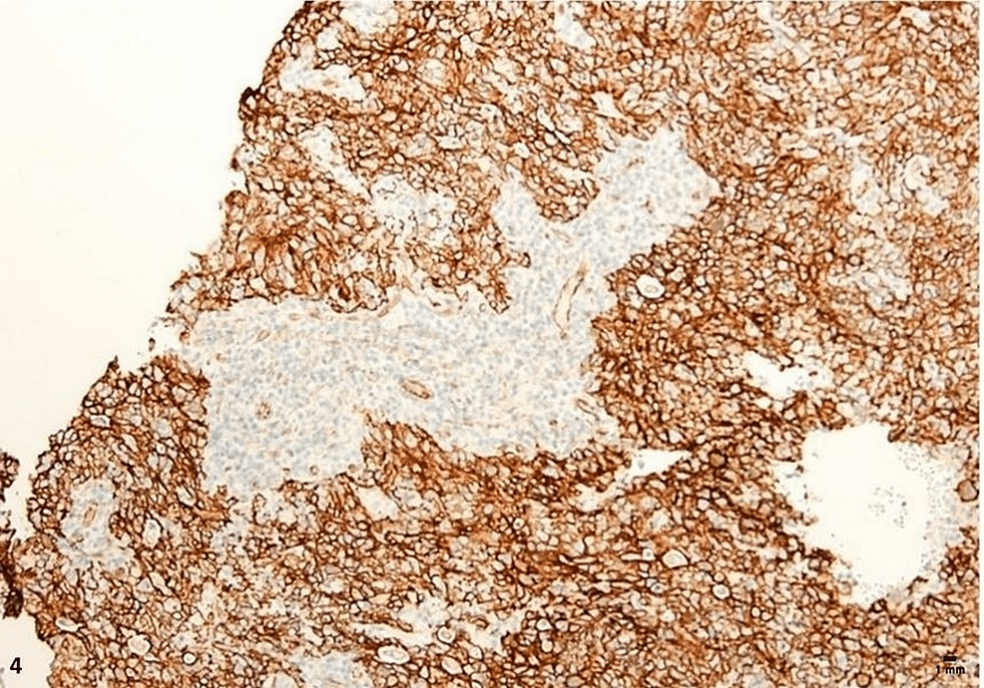
Expression of β-catenin in breast cancer cells. Some of the cancer cells have lost their membranous expression and gained cytoplasmic expression (grade 2 AEB). AEB: abnormal β-catenin expression

**Figure 5:**
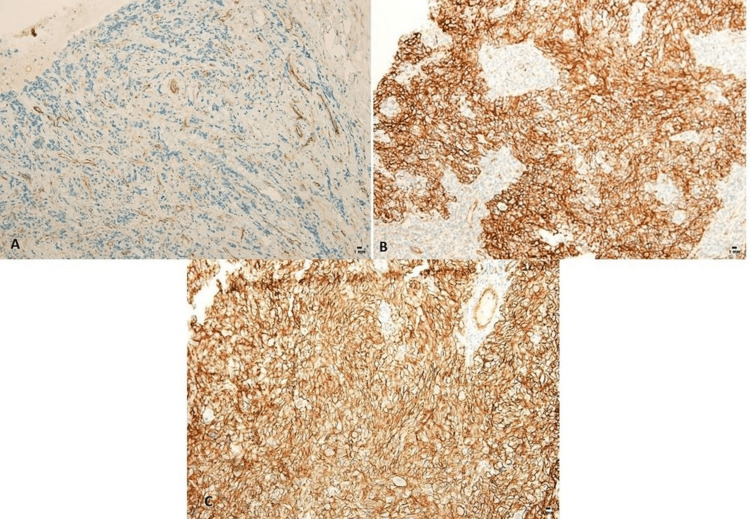
A: Complete loss of membranous expression of β-catenin in a TNBC tumour. (Grade 1 of AEB. Please note scattered claudin-1 residual staining in endothelial cells); B: A TNBC with areas with complete loss of β-catenin staining, while other regions show gain of heavy cytoplasmic expression (Grade 2 of AEB); C: TNBC showing gain of nuclear expression of β-catenin, with heavy cytoplasmic expression (Grade 3 of AEB) AEB: Abnormal Expression of β-catenin; TNBC: Triple negative breast cancer

**Figure 6:**
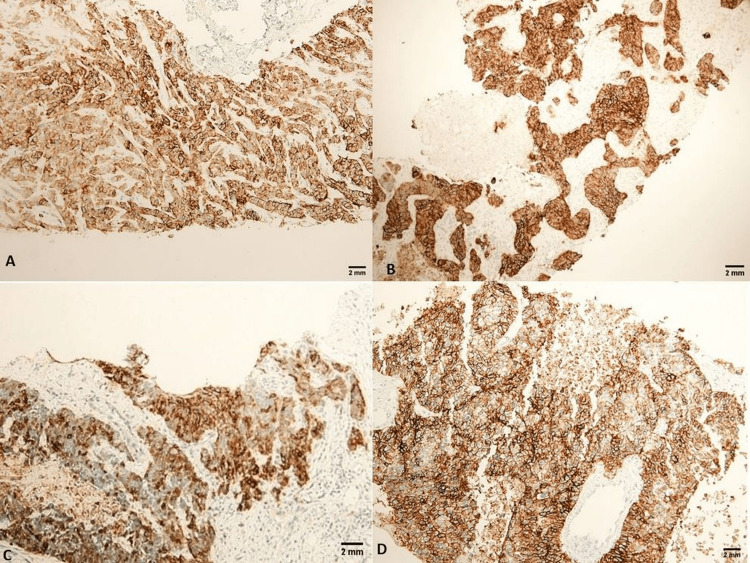
A-D: All four tumours exhibit heavy cytoplasmic and nuclear claudin-1 expression in all cells (IRS of 12/12). IRS: immunoreactivity scoring system

**Figure 7:**
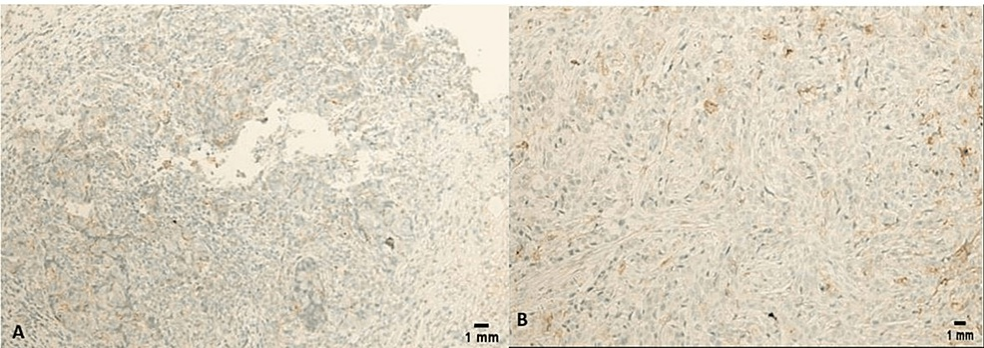
A-B: Two TNBC tumours exhibiting very weak or absent expression of claudin-1 (IRS less than 6). IRS: immunoreactivity scoring system, TNBC: Triple negative breast cancer

Fifty-two TNBC patients were included in the study, with a mean age of 52 years (range of 19-84 years). The location of the expression of claudin-1 in benign breast tissue was apical, membranous, and weak (IRS# 1) (Figure 1). In TNBC cases, on the other hand, 42/52 cases were positive for claudin-1 (81%, χ2=13.705; p<0.001) (Table 1). All claudin-1-positive TNBC cases (100% of cases, 42/42) expressed claudin-1 in the cytoplasm and membranes of their cells (Figure 2, Table 1). Among the claudin-1 positive cases, 20 patients received neoadjuvant chemotherapy, only four achieved pCR (Table 2). β-catenin’s expression in normal breast epithelial cells was membranous and mild in intensity (Figure 3). Most TNBC cases had grade 2 β-catenin expression (77.5%) (Table 3, Figure 4). Figure 5 exhibits the three grades of AEB. Positive expression for claudin-1 was correlated with that of β-catenin (χ2= 23.757; p<0.001) (Table 3). Figure 6 exhibits claudin-1 positive TNBC cases expressing the protein in both the cytoplasm and nuclei. Figure 7 shows two claudin-1 negative (low) TNBC cases.

**Table 1:** Claudin-1 expression in TNBC tissue and its subcellular location. *Statistical significance, CLDN1: Claudin-1; NEG: Negative; POS: Positive; TNBC: Triple negative breast cancer

CLDN1 expression and location in cancer cell	All cases, n	%	CDLN protein expression		
			x^2^	df	P-value
Subcellular localization of CLDN in TNBC tissues					
Pure membranous	0	0			
Cytoplasmic/membranous	42	100			
CLDN1 Score			13.705	1	<0.001*
NEG	10	19			
POS	42	81			

**Table 2:** Claudin-1 expression in TNBC tissue and its subcellular location. *Statistical significance; TNBC: Triple negative breast cancer; SBR: Nuclear grading system of breast cancer; NA: No. of cases (patients) not reported in that parameter; DCIS: Ductal carcinoma in-situ; T: Size of Tumour Mass; N: Presence of Lymph node metastases; M: Presence of Distant Metastases; AWD: Alive with disease; AWOD: Alive without disease; pCR: Pathological complete response; Initial: Before treatment.

Clinic/Path parameters	All cases	%	Significance		
			x^2	df	P-value
Sentinel lymph node status			6.513	1	0.01*
Positive	31	74			
Negative	11	26			
Age			1.597	1	0.206
<50	26				
>50	16				
Right Vs. Left Breast			1.02	1	0.312
Right	17	41			
Left	25	59			
Tumour type (2 patients’ data are NA)					
IDC					
Yes	40	100			
No	0	0			
SBR (Nuclear Grade)			48.55	2	<0.001*
1	0	0			
2	2	5			
3	40	95			
Tumour size (cm, 2 patients’ data are NA)			0.254	1	0.6
<3	23	54			
>3	19	46			
DCIS Presence			1.597	1	0.2
Absent	26	62			
Present	16	38			
Initial Clinical Stage (one patient data is NA)			10.291	3	0.016*
I	3				
II	18				
III	13				
IV	7				
Initial T			13.99	3	0.002*
T1	6				
T2	23				
T3	6				
T4	7				
Initial N (one patient data is NA)			9.71	3	0.02*
N0	17				
N1	12				
N2	10				
N3	2				
Initial M (one patient data is NA)			12.454	1	<0.001*
M0	34				
M1	7				
Survival Data (two patients’ data are NA)			19.899	2	<0.001*
Dead	8				
AWD	3				
AWOD	29				
pCR (Remission achieved)			5	1	0.025*
NO	16	80			
YES	4	20			

**Table 3:** Association between claudin-1 and β-catenin expression in TNBCs. *: Statistical significance; AEB: Abnormal Expression of β-catenin; TNBC: Triple negative breast cancer

Clinical/Path parameters	All cases (n) of AEB in Claudin-1+ cases	%	Significance		
			x^2	df	P-value
β-catenin Score			23.757	2	<0.001*
Grade 1	5	12.5			
Grade 2	31	77.5			
Grade 3	4	10			

Claudin-1 expression and clinical and pathological attributes of TNBC

The correlation between claudin-1 expression and clinical-pathological attributes was further assessed in TNBC tissues (Table 2). Claudin-1 positive expression was correlated with positive sentinel lymph node (χ2=6.513; p=0.01); higher grade (χ2=48.55; p<0.001), higher initial clinical stage (χ2=10.291; p=0.016), higher T (χ2=13.99; p=0.002), N (χ2=9.71; p=0.02), M (χ2=12.454; p<0.001), adverse survival outcome (χ2=19.899; p<0.001), invasive tumour (IDC) diagnosis (100% of cases, 40/40) and failure to achieve pCR (χ2=5, p=0.025). There was no significant association between claudin-1 expression and other criteria, including age (p=0.206), right vs. left breast, (p=0.312), presence of DCIS (p>0.05) and tumour size (p>0.05) (Table 2).

Association of claudin-1 expression and pCR

The pCR term is defined as absence of invasion, and absence of *in situ* residuals in breast and nodes after preoperative radiotherapy or concurrent chemoradiotherapy. Recent work [18] had reported that pCR is a valuable biomarker in categorizing breast cancer patients into favorable and unfavorable groups. In our study, we assessed the relationships between pCR and claudin-1 expression (Table 2). Patients whose tumours expressed claudin-1 were significantly less likely to achieve clinical remission following NAC and more likely to have residual tumours (χ2= 5, p= 0.025) (Table 2).

Association between claudin-1 and EMT markers (β-catenin)

Fifty (50/52) TNBC cases had AEB. The majority of those cases were of Grade 2 AEB which is defined as membranous expression loss with a switch to expression in the cytoplasm. TNBC cases with claudin-1 positive expression were significantly correlated with aberrant expression of β-catenin (χ2= 23.757; p<0.001) (Table 3). This expression was distributed as follows: 1- Loss of membranous expression (Grade 1: LOM, 12.5% of cases, Figure 5A); 2- Membranous expression loss with a switch to cytoplasmic expression (Grade 2, 77.5%, Figure 5B); and 3- Membranous expression loss with a switch to a nuclear expression of the protein, with or without cytoplasmic expression (Grade 3, 10%, Figure 5C). 

“Claudin-1-low” TNBC

Only 10 cases of our sample did not express the claudin-1 protein. Table 4 lists all the clinical, pathologic and demographical data related to these cases. No correlation could be found between loss of claudin-1 and clinical stage, TNM stage, sentinel lymph node status, adverse survival outcome, pathological complete response, DCIS presence, tumour size, right versus left breast, age and concurrent β-catenin expression. Only SBR nuclear grade (p=0.032) and invasive phenotype (all 10 cases were IDC) showed positive correlation. The majority of claudin-1 low TNBC cases were T2 (7/10), M0 (8/10), AWOD (7/10), AEB grade 2 (7/10), tumour size >3 cm (8/10) and had absent DCIS (8/10). 

**Table 4:** Claudin-1 low cases clinical, pathological and immunohistochemical attributes. *Statistical significance; TNBC: Triple negative breast cancer; SBR: Nuclear grading system of breast cancer; DCIS: Ductal carcinoma in-situ; T: Size of Tumour Mass; N: Presence of Lymph node metastases; M: Presence of Distant Metastases; AWD: Alive with disease; AWOD: Alive without disease; pCR: Pathological complete response; Initial: Before treatment; AEB: Abnormal expression of β-catenin.

Clinic/Path parameters	All cases	%	Significance		
			x^2	df	P-value
Sentinel lymph node status			0.424	1	>0.05
Positive	7	70			
Negative	3	30			
Age (years)			0	1	>0.05
<50	5				
>50	5				
Right Vs. Left Breast			0.017	1	>0.05
Right	4	40			
Left	6	60			
Tumour type					
IDC					
Yes	10	100			
No	0	0			
SBR (Nuclear Grade)			6.838	2	0.032*
1	0	0			
2	1	10			
3	9	90			
Tumour size (cm)			1.406	1	>0.05
<3	2	20			
>3	8	80			
DCIS Presence			1.406	1	>0.05
Absent	8	80			
Present	2	20			
Initial Clinical Stage			4.688	3	>0.05
I	0				
II	5				
III	5				
IV	0				
Initial T			5.223	3	>0.05
T1	0				
T2	7				
T3	2				
T4	1				
Initial N			0.507	3	>0.05
N0	2				
N1	4				
N2	1				
N3	3				
Initial M			1.406	1	>0.05
M0	8				
M1	2				
Survival Data			2.457	2	>0.05
Dead	1				
AWD	2				
AWOD	7				
pCR (Remission achieved)			0.024	1	>0.05
NO	4	57			
YES	3	43			
β-catenin score	Total no of AEB+ in [claudin-1 low] cases		2.457	2	>0.05
Grade 1	2				
Grade 2	7				
Grade 3	1				

## Discussion

TNBCs positive expression of claudin-1

Our study shows that TNBC patients have significant expression of claudin-1, and that this expression correlates with invasive phenotype, sentinel lymph node positivity, higher tumour grade, higher clinical and TNM stage, and adverse survival outcome. “Claudin-1 low” patients were the minority in our study, with no significant association except for the grade of the tumours (Table 4). Claudin-1 was first described as an integral part of the tight junction core structure in the 1990s [19]. Since then, claudin-1 has been found to be over-expressed in various types of human cancers. [7]. In breast cancer in general, claudin-1 is hypothesized to play a double role, first as a tumour suppressor on one hand [12,20], where its downregulation resulted in breast cell tumourigenesis. In the rest of cancers of the breast, claudin-1 was found to play the role of a tumour enhancer/facilitator [21-22]. In line with the latter studies [21,22], the tumours in our sample exhibited strong claudin-1 expression in the membranes, cytoplasm and nuclei of the tumours’ sample (Figure 6A-6D). This cytoplasmic accumulation may have led to disruption of the tight junction strands, allowing for an unchecked exposure of the tissues lying underneath the paracellular seal, and may also have facilitated the process of transformed cell migration and invasion [7].

Correlation of Aberrant Expression of Claudin-1 and β-catenin in Samples of Our Patient Cohort

Our study results also indicate a correlation between claudin-1 and β-catenin expressions. Claudin-1 is a downstream target of β-catenin [23]. β-catenin is a major component of the EMT event. EMT has been identified as a critical step in the formation of secondary metastases in many tumours including renal [24], choriocarcinoma [25], melanoma [26], endometrial [27], prostate [28], thyroid [29], colon [30], liver [31], gastric [32], and breast cancers [33-34]. In normal state, cells are held tightly together by a cytoskeleton comprised of many components including adherens junctional proteins, and tight junction proteins including the claudin proteins. β-catenin works as an adaptor protein in this process, linking the intra-cytoplasmic domain of E-cadherin to the cytoskeleton of the cell [23,35]. Thus, the membranous expression of β-catenin, claudins and E-cadherin is pivotal in maintaining cell polarity [23,35], which, in turn, is critical to keeping intact cellular signal transduction and functions. From The disrupted expression of two chief components of the cytoskeleton in TNBCs, i.e, claudin-1 and β-catenin, may play role in cellular transformation. 

EMT, β-catenin and Claudin-1

β-catenin is a well-known member of the Wingless/(Wnt) signalling pathway. Recent studies have shown that AEB represents the activated state of protein, and that β-catenin expression was an independent factor for breast cancer survival [16,36,37]. In our study, we report a switch in the expression of both proteins, claudin-1 and β-catenin, from normal membranous positions to the cytoplasm of the TNBC cases, and in some cases to the nuclei (Figure 5B-5C for β-catenin and Figure 6A-6D for claudin-1). While claudin-1’s role as a proponent of cell migration, invasion and the EMT phenomenon is well documented in other cancers [38], in breast cancer there is evidence that claudin-1 may have a dual role, first as a target for one or more of the EMT pathway molecules such as β-catenin and Slug/Snail-1 [20], or secondly as an effector altering the expression of EMT-related molecules either by transcription factors’ activation such as slug and zeb1 or through modulation of the β-catenin/Tcf signaling pathway [16].

Claudin-1 and Pathological Complete Response (pCR)

pCR following NAC has been singled out as a valuable prognostic indicator for local control and survival [18]. In our claudin-1 positive patients, 20 patients received pre-surgical, NAC treatment, while the rest did not receive neoadjuvant treatment, but received different modalities of surgical resection. From the 20 claudin-1 positive TNBC patients who received the NAC, a statistically significant number (p=0.025) of those patients (16/20 in total) failed to achieve pCR evident by residual tumours’ presence (Table 2). This is the first report of the relationship between failure to achieve pCR and claudin-1 positivity in TNBC patients. In our opinion, this may add more weight to the value of claudin-1 testing in TNBC patients. Further studies are needed to analyze the nature of this relationship in-depth.

Our results suggest a correlation in the expression levels of claudin-1 in TNBC and clinical surrogates of carcinogenesis and cancer progression, thus this study makes the case for a claudin-1 high subset in TNBC patients. Earlier, fewer studies have shown similar results [20-22]. A precedent of this dual role for claudin-1 seen above in TNBC patients was also reported recently [39] in colorectal cancer patients. In the colon, claudin-1 plays a dual role as a tumour-suppressor gene and as an oncogene [39]. 

Limitations

A possible limitation to our study is the small number of patients. A larger study is in order to confirm our results, and to assess the "claudin-1 low" subgroup. A future analysis to assess the correlation between claudin-1 nucleic vs. protein expression in TNBC is needed as well. 

## Conclusions

The above presents a complex role of claudin-1 in TNBC patients. In our study, claudin-1 expression is correlated with invasion, sentinel lymph node metastases, higher tumour grade, higher clinical and TNM stage, and adverse clinical outcome in TNBC patients. Furthermore, evidence of statistical and morphological correlation between claudin-1 and β-catenin expressions was presented, providing further indication about the role of claudin-1 in TNBC tumours. Collectively, this may suggest the need for further mechanistic studies to illustrate the exact role of this protein in TNBC patients and to address possible ways to modulate this expression to push back against the aggressive nature of TNBCs. This study also showed that "claudin-1 low" TNBC group appears to be the minority of patients in this category, contrary to the majority of studies analyzing TNBC expression of claudin-1. And finally, we have presented possible use of claudin-1 in predicting pCR, a recent important prognostic indicator of breast cancer. A larger cohort may be needed to validate the above. 
